# Undiagnosed abnormal postpartum blood loss: Incidence and risk factors

**DOI:** 10.1371/journal.pone.0190845

**Published:** 2018-01-10

**Authors:** Aude Girault, Catherine Deneux-Tharaux, Loic Sentilhes, Françoise Maillard, François Goffinet

**Affiliations:** 1 INSERM UMR 1153, Obstetrical, Perinatal and Paediatric Epidemiology Research Team (Epopé), Centre for Epidemiology and Statistics Sorbonne Paris Cité (CRESS), DHU Risks in pregnancy, Paris Descartes University, Paris, France; 2 Department of Obstetrics and Gynaecology, Angers University Hospital, France; 3 Port-Royal Maternity Unit, Department of Obstetrics Paris, Cochin Broca Hôtel-Dieu hospital, Assistance Publique-Hôpitaux de Paris, Paris, France; Public Library of Science, UNITED KINGDOM

## Abstract

**Background:**

We aimed to evaluate the incidence of undiagnosed abnormal postpartum blood loss (UPPBL) after vaginal delivery, identify the risk factors and compare them to those of postpartum haemorrhage (PPH).

**Method:**

The study population included women who participated in a randomized controlled trial of women with singleton low-risk pregnancy who delivered vaginally after 35 weeks’ gestation (n = 3917). Clinical PPH was defined as postpartum blood loss ≥ 500 mL measured by using a collector bag and UPPBL was defined by a peripartum change in haemoglobin ≥ 2 g/dL in the absence of clinical PPH. Risk factors were assessed by multivariate multinomial logistic regression.

**Results:**

The incidence of UPPBL and PPH was 11.2% and 11.0% of vaginal deliveries, respectively. The median peripartum change in Hb level was comparable between UPPBL and PPH groups (2.5 g/dL interquartile range [2.2–3.0] and 2.4 g/dL IQR [1.5–3.3]). Risk factors specifically associated with UPPBL were Asian geographical origin (adjusted OR [aOR] 2.3, 95% confidence interval [CI] 1.2–4.2; p = 0.009), previous caesarean section (aOR 3.4, 2.1–5.5; p<0.001) and episiotomy (aOR 2.6, 1.8–3.6; p<0.001). Risk factors for both UPPBL and PPH were primiparity, long duration of labour, instrumental delivery and retained placenta.

**Conclusion:**

Undiagnosed abnormal postpartum blood loss is frequent among women giving birth vaginally and has specific risk factors. The clinical importance of this entity needs further confirmation, and the benefit of systematic or targeted prevention strategies needs to be assessed.

## Introduction

Postpartum haemorrhage (PPH) is defined by blood loss ≥ 500 mL within 24 hours after delivery[1]. It complicates 5% to 15% of deliveries and remains a major component of severe maternal morbidity and maternal mortality[1–4]. However, a few reports have suggested that women may present peripartum blood count changes reflecting abnormal blood loss, ≥ 500 mL, although no PPH has been diagnosed clinically, with reported incidences as high as 13% [5,6].

This undiagnosed abnormal postpartum blood loss (UPPBL) has potential adverse consequences that could justify investigating their incidence and risk factors. Indeed, even though UPPBL is probably not a cause of severe maternal morbidity, it may have short- and long-term adverse effects on maternal and child health because of the induced and most often uncorrected postpartum anaemia [7–10]. Women with untreated postpartum anaemia, even moderate, experience more breathlessness, heart palpitations, fatigue and infections [11] and are at increased risk of maternal stress, anxiety, emotional instability, postpartum depression and reduced cognitive performance [7–10] than women without anaemia, up to 9 months after delivery. In addition to these consequences on maternal health, postpartum anaemia also affects mother–child interactions, compromises the mother–child bond and is associated with impaired infant development at 9 months of age [10].

A better knowledge of the epidemiology of UPPBL could allow for estimating its clinical significance and eventually investigating its causes and consequences. Our main hypothesis is that women with UPPBL have delayed excessive bleeding that is not easily assessed during the rigorous monitoring period in the labour ward or during the first 24 hours of routine surveillance. A central issue is to determine whether UPPBL risk factors are specific to UPPBL or are shared with PPH.

The TRAction of the CORd (TRACOR) trial [12], which tested the impact of controlled cord traction on the incidence of PPH, offers the opportunity to study UPPBL. Indeed, in this trial, the management and monitoring of the third stage of labour was standardized, including the placement of a collector bag after delivery, which allows for standardizing and optimizing the measurement of blood loss in the immediate postpartum period [13–15], and blood count performed before and after delivery for all women. This protocol allowed for developing a clear and exhaustive definition of both PPH and UPPBL.

The objective of this study was to estimate the incidence of UPPBL, identify its risk factors and compare them to those of PPH in women delivering vaginally.

## Materials and methods

The study population included women who participated in the TRACOR trial [12] (ClinicalTrials.gov identifier NCT01044082). This multicentre randomized controlled trial with two parallel groups was conducted in five French university hospitals between 2010 and 2011 and showed no impact of controlled cord traction on the incidence of PPH, defined by blood loss ≥ 500 mL measured by using a graduated collector bag. The two groups did not differ in secondary outcomes, including mean peripartum changes in haemoglobin (Hb) level and haematocrit (Ht) value. Except for controlled cord traction, the active management of the third stage of labour and of PPH if it occurred was standardized in the five study centers, following national and international guidelines [1,15,16], and included: a 5-UI oxytocin intravenous injection after shoulder delivery, clamping and cutting of the cord within two minutes of birth; placement of a graduated (100 mL graduation) collector bag (MVF Merivaara France) just after birth, left in place until the birth attendant judged that bleeding had stopped and that there was no reason to monitor further, and always at least for 15 minutes; and manual removal of the placenta at 30 minutes after birth if not expelled. If PPH occurred, the management strategy in the 5 study centers was standardized following the national guidelines.

The trial population consisted of women > 18 years old who vaginally delivered singletons after 35 weeks’ gestation (n = 4058). Women with coagulation disorders, placenta prævia, in utero foetal death, and multiple gestation and those who did not understand French were excluded. For our study, we excluded women who did not have a collector bag placed immediately after delivery (n = 45) and did not have blood samples before delivery or at day 2 post-delivery (n = 98), because the diagnosis of PPH or UPPBL (see definitions below) was not possible for these women. Our study sample consisted of 3917 women.

The principal outcome was the occurrence of PPH or UPPBL. PPH was defined by blood loss ≥ 500 mL measured at the removal of the collector bag and/or any second-line treatment for PPH (second-line uterotonic agent, transfusion, intrauterine tamponade, uterine artery embolization, surgery). As previously proposed [6,17,18], UPPBL was defined as a change in peripartum Hb level of at least 2 g/dL without any clinical diagnosis of PPH calculated as the difference between the last Hb measurement during pregnancy and at day 2 postpartum. Prepartum Hb was measured between week 33 of gestation and before arrival at the labour ward for 91.1% of women, at arrival at the labour ward for 2.1%, and between weeks 20 and 33 of gestation for 6.8%. A sensitivity analysis involved data for women who had a blood test within the two weeks before delivery (n = 1226).

In a secondary analysis, we tested three other definitions of UPPBL used in previously published studies (all definitions selected only women with no PPH): peripartum Hb level change of at least 2 g/dL associated with postpartum Hb level ≤ 10 g/dL [16,19]; peripartum change in Ht value ≥ 5% calculated as the difference between Ht value before delivery and at day 2 postpartum [13,20]; and blood loss ≥ 500 mL calculated with a formula proposed to quantify perioperative blood loss: ((peripartum Ht change) x total blood volume (TBV))/ 0.35, where TBV (mL) = weight (in kg) x 65 x 1.4 [21,22].

The following data were collected for each woman: the pre-existing characteristics geographical origin, age, body mass index (BMI), parity (primipara, multipara with no previous caesarean section, multipara with previous caesarean section), history of PPH, pre-existing chronic disease (≥ 1 of high blood pressure, diabetes, autoimmune disease), and smoking before pregnancy; the pregnancy characteristics smoking during pregnancy, polyhydramnios, weight gain during pregnancy and hypertensive disorder during pregnancy (gestational hypertension or preeclampsia); and the labour and delivery characteristics gestational age at delivery, induction of labour, total dose of oxytocin received during labour, epidural anaesthesia, hyperthermia during labour, duration of labour, duration of expulsive effort, mode of delivery (spontaneous, forceps, vacuum or spatula), type of perineal trauma (none, episiotomy with or without first- and second-degree tear, first- and second-degree tear without episiotomy, third- and fourth- degree tear), retained placenta, birth weight and maternity unit.

These characteristics were tested as risk factors for UPPBL and PPH by univariate analysis, using Pearson’s χ^2^ test or Fisher’s exact test when appropriate for nominal variables, and Student’s t test for continuous variables. A multivariate multinomial logistic regression was then performed to calculate adjusted ORs (aORs) with their 95% confidence intervals (CIs), with the dependent variable in three classes (PPH, UPPBL, no UPPBL or PPH). Variables included in multivariable regression analysis were chosen from the literature and from the results of univariate analysis. After having tested that the quantitative variables had a linear relationship with the dependent variable, all variables except total dose of oxytocin received during labour were entered in the multivariate model as continuous variables. The total dose of oxytocin received during labour was included as a categorical variable by quartiles. Two clinically relevant interactions were tested: duration of labour and oxytocin dose, instrumental delivery and episiotomy. Secondary analyses involved analysis by the other definitions for UPPBL with the same strategy.

Data for 150 women (3.9%) were not included in the multivariate analysis because of missing data for at least one selected factor, so this analysis represented data for 96.1% of our initial study population. This population was comparable to the analysed population for all characteristics (S1 Table).

P<0.05 was considered statistically significant. Given our sample size, with a two-sided significance level of 5%, the available power was at least 70% to show OR of at least 2.0 for any studied characteristic. Analyses involved use of STATA v12.1 (StataCorp, College Station, TX).

## Results

Among the 3917 women included in our analysis, 439 had UPPBL (11.2%, 95% CI 10.2–12.2) and 430 had PPH (401 had blood loss ≥ 500 mL in the bag, 29 received second-line uterotonics for PPH although the measured blood loss was at that moment <500 mL) (11.0%, 95% CI 10.0–12.0). The women with and without cord traction were equally distributed in the UPPBL group (respectively 49.3% and 50.7%). The median peripartum change in Hb level was comparable for the UPPBL and PPH groups (respectively 2.5 g/dL IQR [2.2–3.0] and 2.4 g/dL IQR [1.5–3.3], respectively) but not the control group (0.5 g/dL IQR [0–1.1]) (Table 1). The rate of women with postpartum anemia (Hb level ≤ 10 g/dL) was comparable for the UPPBL and PPH groups (respectively 56.5% and 58.1%, Pearson p = 0.4) but lower in the control group (9.7%). The median blood loss at bag removal was increased in the PPH group, as expected, given the definition criteria for the three groups (Table 1). The median duration between delivery and removal of collector bag differed between UPPBL, PPH and control groups (30 min IQR [22–42], 37 min IQR [28–49], and 23 min IQR [15–33] min, respectively, (p<0.001, nonparametric k-sample test). The characteristics of women, labour and delivery are in Table 2 and those associated with UPPBL and PPH on univariate analysis are in Table 3.

**Table 1:** Prepartum, postpartum and median peripartum change in haemoglobin (Hb) level, number of women with postpartum anemia and blood loss in women with undiagnosed abnormal postpartum blood loss (UPPBL), postpartum haemorrhage (PPH) and controls.

	UPPBL^a^ (n = 439)	PPH^b^ (n = 430)	Control^c^ (n = 3048)
Measured blood loss at bag removal (mL)	200 [110–350]	600 [500–800]	100 [50–200]
Peripartum change in Hb level (g/dL)	2.5 [2.2–3.0]	2.4 [1.5–3.3]	0.5 [0–1.1]
Prepartum Hb level (g/dL)	12.5 [11.8–13.3]	12.1 [11.3–12.8]	12.0 [11.3–12.7]
Postpartum day two Hb level (g/dL)	9.8 [9.1–10.7]	9.6 [8.7–10.6]	11.6 [10.7–12.4]
Postpartum Hb level ≤ 10g/dL, n(%)	248 (56.5)	250 (58.1)	300 (9.7)

Data are median [interquartile range (IQR)] or n(%) if specified

^a:^ UPPBL defined by peripartum change in haemoglobin level ≥ 2 g/dL with no PPH diagnosed

^b:^ Defined by a measured blood loss ≥500 mL in the collector bag and/or any second line treatment for PPH

^c:^ neither PPH nor UPPBL

**Table 2:** Characteristics of the study population (n = 3917).

Characteristics of women, labour and delivery	n	(%)
**Geographic origin**		
**France**	**3198**	**(81.6)**
**Europe**	**142**	**(3.6)**
**North Africa**	**248**	**(6.3)**
**Sub-Saharan Africa**	**117**	**(3.0)**
**Asia**	**71**	**(1.8)**
**French overseas territories and departments**	**52**	**(1.3)**
**Other origins**	**73**	**(1.9)**
**Age (year) median, [IQR]**	**30**	**[26–34]**
**BMI**^a^ (**kg/m^2^**) **median, [IQR]**	**21.8**	**[20.0–24.3]**
**Parity**		
**Primipara**	**2110**	**(53.9)**
**Multipara with no previous caesarean**	**1568**	**(40.0)**
**Multipara with previous caesarean**	**237**	**(6.1)**
**History of clinical PPH**	**79**	**(2.0)**
**Pre-existing chronic disease**^b^	**92**	**(2.3)**
**Smoking before pregnancy**	**1134**	**(29.0)**
**Smoking during pregnancy**	**635**	**(16.2)**
**Polyhydramnios**	**28**	**(0.7)**
**Weight gain during pregnancy (kg) median, [IQR]**	**13**	**[10–16]**
**Hypertensive disorder during pregnancy**^c^	**61**	**(1.6)**
**Gestational age at delivery (weeks) median, [IQR]**	**40**	**[39–41]**
**Induction of labour**	**750**	**(19.1)**
**Total dose of oxytocin (mUI) median, [IQR]**	**1088**	**[452–2430]**
**Epidural anaesthesia**	**3799**	**(97.0)**
**Hyperthermia during labour**	**181**	**(4.6)**
**Duration of labour (h) median, [IQR]**	**3.5**	**[2.3–5.0]**
**Duration of expulsive efforts (min) median, [IQR]**	**13**	**[8–23]**
**Mode of delivery**		
**Spontaneous**	**3195**	**(81.6)**
**Forceps**	**265**	**(6.8)**
**Vacuum**	**276**	**(7.1)**
**Spatula**	**179**	**(4.6)**
**Type of perineal trauma**		
**None**	**1048**	**(26.8)**
**Episiotomy ± 1st- and 2nd-degree tear**	**996**	**(25.4)**
**1st- and 2nd-degree tear without episiotomy**	**1819**	**(46.4)**
**3rd and 4th degree tear**	**54**	**(1,4)**
**Retained placenta**	**185**	**(4.7)**
**Birth weight (g) median, [IQR]**	**3360**	**[3090–3660]**
**Maternity unit**		
**1**	**926**	**(23.7)**
**2**	**382**	**(9.7)**
**3**	**640**	**(16.3)**
**4**	**888**	**(22.7)**
**5**	**108**	**(27.6)**

Data are n(%) unless indicated.

^a:^ BMI: body mass index (weight (kg)/height^2^ (m))

^b:^ ≥ 1 among high blood pressure, diabetes, and autoimmune disease

^c:^ Gestational hypertension or preeclampsia during pregnancy

**Table 3:** Univariate analysis of characteristics of women, labour and delivery associated with UPPBL, clinical PPH, and controls.

Characteristics of women, labour and delivery	Control n = 3048 (77.8%) n (% in line)	UPPBL n = 439 (11.2%) n (% in line)	PPH n = 430 (11.0%) n (% in line)	OR_UPPBL/control_ 95% CI	p	OR_PPH/control_ 95% CI	p
Geographical origin					**0.001**		0.2
France	2511 (78.5)	347 (10.9)	340 (10.6)	Ref.		Ref.	
Europe	113 (79.6)	14 (9.9)	15 (10.6)	0.9 (0.5–1.6)		1.0 (0.6–1.7)	
North Africa	193 (77.8)	21 (8.5)	34 (13.7)	0.8 (0.5–1.2)		1.3 (0.9–1.9)	
Sub Saharan Africa	88 (75.2)	14 (12.0)	15 (12.8)	1.1 (0.6–2.0)		1.3 (0.7–2.2)	
Asia	41 (57.7)	22 (31.0)	8 (11.3)	3.9 (2.3–6.6)		1.4 (0.7–3.1)	
French overseas territories and departments	41 (78.8)	6 (11.5)	5 (9.6)	1.0 (0.4–2.5)		0.9 (0.3–2.3)	
Other origins	47 (64.4)	13 (17.8)	13 (17.8)	2.0 (1.0–3.7)		2.0 (1.1–3.8)	
Age (year) median, [IQR]	30 [26–34]	30 [26–34]	30 [27–34]		0.8		0.7
BMI^a^ (kg/m^2^) median, [IQR]	21.8 [20.0–24.2]	21.6 [19.7–24.0]	22.2 [20.2–24.9]		0.3		**0.02**
Parity					<**0.001**		<**0.001**
Primipara	1500 (71.1)	333 (15.8)	277 (13.1)	4.6 (3.5–6.0)		2.0 (1.6–2.6)	
Multipara with no previous caesarean	1377 (87.8)	67 (4.3)	124 (7.9)	Ref.		Ref.	
Multipara with previous caesarean	171 (71.3)	39 (16.5)	29 (12.2)	4.7 (3.1–7.3)		1.9 (1.2–2.9)	
History of clinical PPH	57 (72.1)	4 (5.1)	18 (22.8)	0.5 (0.2–1.3)	0.2	2.3 (1.3–3.9)	**0.003**
Pre-existing chronic disease^b^	74 (80.4)	6 (6.5)	12 (13.0)	0.6 (0.2–1.3)	0.2	1.1 (0.6–2.1)	0.6
Smoking before pregnancy	901 (79.4)	124 (11.0)	109 (9.6)	0.9 (0.7–1.2)	0.5	0.8 (0.6–1.0)	0.06
Smoking during pregnancy	523 (82.4)	53 (8.3)	59 (9.3)	0.7 (0.5–0.9)	**0.007**	0.8 (0.6–1.0)	0.07
Polyhydramnios	19 (67.9)	3 (10.7)	6 (21.4)	1.1 (0.3–3.7)	0.9	2.3 (0.9–5.7)	0.08
Weight gain (kg) median, [IQR]	13 [10–16]	13 [10–16]	14 [10–17]		0.3		0.2
Hypertensive disorder during pregnancy^c^	42 (68.9)	7 (11.5)	12 (19.6)	1.1 (0.5–2.6)	0.8	2.0 (1.1–3.9)	**0.03**
Gestational age at delivery (weeks) median, [IQR]	40 [39–40]	40 [39–40]	40 [39–41]		0.3		<**0.001**
Induction of labour	557 (74.3)	86 (11.5)	107 (14.3)	1.1 (0.9–1.4)	0.4	1.5 (1.1–1.9)	**0.001**
Total dose of oxytocin (mUI) median, [IQR]	975 [425–2187]	1407 [538–3263]	1725 [638–3750]		<**0.001**		<**0.001**
Epidural anaesthesia	2956 (77.8)	425 (11.2)	418 (11.0)	0.9 (0.5–1.7)	0.8	1.1 (0.6–2.0)	0.8
Hyperthermia during labour	113 (62.4)	42 (23.2)	27 (14.4)	2.7 (1.9–4.0)	<**0.001**	1.7 (1.1–2.6)	**0.02**
Duration of labour (h) median, [IQR]	3.3 [2.2–4.7]	4.3 [3.2–5.8]	4.3 [2.9–5.6]	<**0.001**			<**0.001**
Duration of expulsive efforts (min) median, [IQR]	12 [7–20]	18 [10–30]	19 [10–30]		<**0.001**		<**0.001**
Mode of delivery					<**0.001**		<**0.001**
Spontaneous	2616 (81.9)	293 (9.2)	286 (8.9)	Ref.		Ref.	
Forceps	148 (55.9)	59 (22.3)	58 (21.9)	3.6 (2.6–4.9)		3.6 (2.6–5.0)	
Vacuum	205 (74.3)	29 (10.5)	42 (15.2)	1.3 (0.8–1.9)		1.9 (1.3–2.7)	
Spatula	79 (44.1)	57 (31.8)	43 (24.0)	6.4 (4.5–9.2)		5.0 (3.4–7.4)	
Type of perineal trauma					<**0.001**		<**0.001**
None	886 (84.5)	61 (6.1)	101 (10.4)	Ref.		Ref.	
Episiotomy ± 1^st^- and 2^nd^-degree tear	609 (61.1)	244 (24.5)	143 (14.4)	5.8 (4.3–7.8)		2.1 (1.6–2.7)	
1^st^- and 2^nd^-degree tear without episiotomy	1522 (83.7)	127 (7.0)	170 (9.5)	1.1 (0.9–1.7)		1.0 (0.8–1.3)	
3^rd^- and 4^th^-degree tear	31 (57.4)	7 (13.0)	16 (29.6)	3.3 (1.4–7.8)		4.5 (2.4–8.6)	
Retained placenta	105 (56.8)	26 (14.0)	54 (29.2)	1.8 (1.1–2.7)	**0.01**	4.1 (2.9–5.7)	<**0.001**
Birth weight (g) median, [IQR]	3350 [3070–3650]	3320 [3080–3640]	3500 [3200–3760]		0.9		<**0.001**

Data are n(%) unless indicated and odds ratios (ORs) and 95% confidence intervals (95% CIs);

^a:^ BMI: body mass index (weight (kg)/height^2^ (m))

^b:^ ≥ 1 among high blood pressure, diabetes, and autoimmune disease;

^c:^ Gestational hypertension or preeclampsia during pregnancy

Risk factors specifically associated with UPPBL on multivariate analysis were Asian geographic origin (aOR 2.3, 95% CI 1.2–4.2), previous caesarean section (aOR 3.4, 95% CI 2.1–5.5) and episiotomy (aOR 2.6, 95% CI 1.8–3.6) (Table 4). Smoking during pregnancy was associated with reduced risk of UPPBL (aOR 0.7, 95% CI 0.5–0.9). Risk factors for both UPPBL and PPH were primiparity, long duration of labour, instrumental delivery by forceps or spatula, and retained placenta. Risk factors for PPH alone were history of PPH, increased gestational age at delivery, oxytocin dose, long duration of expulsive efforts and increased weight of the newborn.

**Table 4:** Multivariate analysis^a^ of risk factors for UPPBL and PPH (n = 3917).

Characteristics of women, labour and delivery	UPPBL/control aOR^a^ (95% CI)	p	PPH/control aOR^a^ (95% CI)	p
Asian geographic origin	2.3 (1.2–4.2)	**0.009**	1.2 (0.5–2.7)	0.7
Age (year)	1.0 (1.0–1.0)	0.1	1.0 (1.0–1.0)	0.3
BMI^b^ (/5 kg.m^-2^)	1.0 (0.9–1.1)	0.9	1.1 (1.0–1.3)	0.07
Parity		<**0.001**		**0.02**
Primipara	3.3 (2.3–4.6)		1.5 (1.1–2.1)	
Multipara with no previous caesarean	Ref.		Ref.	
Multipara with previous caesarean	3.4 (2.1–5.5)		1.4 (0.9–2.3)	
History of clinical PPH	0.9 (0.3–2.6)	0.8	3.4 (1.8–6.2)	<**0.001**
Smoking during pregnancy	0.7 (0.5–0.9)	**0.02**	0.8 (0.6–1.1)	0.2
Hypertensive disorder during pregnancy^c^	0.8 (0.3–2.0)	0.7	1.6 (0.8–3.3)	0.2
Gestational age at delivery (weeks’ gestation)	1.0 (0.9–1.1)	0.5	1.2 (1.1–1.3)	**0.001**
Induction of labour	1.2 (0.9–1.6)	0.3	1.2 (0.9–1.6)	0.2
Duration of labour (/2 hr)	1.2 (1.1–1.4)	**0.04**	1.1 (1.0–1.1)	0.07
Duration of expulsive efforts (/10 min)	0.9 (0.8–1.1)	0.4	1.1 (1.0–1.3)	**0.04**
Total dose of oxytocin (mUI) during labour		0.2		**0.002**
0	Ref.		Ref.	
0–460	1.3 (0.9–1.8)		1.3 (0.9–2.0)	
461–1650	1.3 (1.0–1.9)		1.3 (0.9–1.7)	
>1650	1.4 (1.0–2.0)		1.9 (1.4–2.7)	
Hyperthermia during labour	1.3 (0.9–2.0)	0.2	0.9 (0.6–1.5)	0.7
Mode of delivery		<**0.001**		<**0.001**
Spontaneous	Ref.		Ref.	
Forceps	2.8 (1.8–4.2)		2.9 (1.9–4.5)	
Vacuum	1.1 (0.7–1.8)		1.4 (0.9–2.1)	
Spatula	1.8 (1.2–2.8)		3.1 (2.0–5.0)	
Type of perineal trauma		<**0.001**		**0.02**
None	Ref.		Ref.	
Episiotomy ± 1^st^- and 2^nd^-degree tear	2.6 (1.8–3.6)		1.0 (0.7–1.3)	
1^st^- and 2^nd^-degree tear without episiotomy	0.9 (0.7–1.3)		0.7 (0.5–1.0)	
3^rd^- and 4^th^-degree tear	1.8 (0.7–4.6)		1.9 (0.9–4.0)	
Retained placenta	2.3 (1.4–3.8)	**0.001**	5.9 (4.0–8.7)	<**0.001**
Birth weight (/500 g)	1.0 (0.9–1.2)	0.5	1.3 (1.1–1.5)	<**0.001**

^a:^ Multivariate model including all the variables listed in the table and the maternity unit

^b:^ BMI: body mass index (weight (kg)/height^2^ (m))

^c:^ Gestational hypertension or preeclampsia during pregnancy

When restricting the definition of UPPBL to women who also had clear postpartum anaemia (Hb level ≤ 10 g/dL), we found an incidence of 6.5% (248/3917) and the same risk factors as those found in the main analysis (S2 Table). The analysis with the other definitions for UPPBL showed an UPPBL incidence of 16.1% for the definition based on peripartum change in Ht ≥ 5% and 19.1% for the definition based on calculated blood loss. The risk factors identified were comparable to those found in the main analysis (S2 Table).

The sensitivity analysis performed among women who had a blood test within the two weeks before delivery (n = 1226, 203 women with UPPBL (16.6%), 148 women with PPH (12.1%) and 875 controls) also found similar risk factors for UPPBL and PPH (S3 Table). The median peripartum change in Hb level in women who had a blood test within the 2 weeks before delivery was comparable for the UPPBL and PPH groups (respectively 2.5 g/dL IQR [2.2–3.0] and 2.7 g/dL IQR [1.5–3.6], respectively).

## Discussion

We found that UPPBL occurred in about 1 in 10 vaginal deliveries and had specific risk factors not associated with PPH: Asian geographical origin, previous caesarean section and episiotomy.

In this large prospective trial, all women had the same active management of the third stage of labour in line with international guidelines [1,15] and a blood count before delivery and at day 2 postpartum, which allowed for a homogenous and exhaustive identification of both PPH and UPPBL. The monitoring of the immediate postpartum period was rigorous because of the trial but corresponds to standard care.

Although our sample originated from a randomized trial, it included a large population of pregnant women with few exclusion criteria; indeed, the distribution of the main characteristics of women and deliveries (age, BMI, parity, previous caesarean section, type of delivery and episiotomy) was similar in our study population and in the French general population of parturients [23]. In addition, based on the literature, the 11.0% incidence of PPH is within the expected range when a collector bag is used [13,24].

One limitation of our study is the variability of the Hb measurement. Indeed, women could have their prepartum and postpartum blood tests performed in two different laboratories; the amount of fluid replacement after delivery, which can be a cause of hemodilution, was not standardized; and the time between the prepartum blood test and delivery was not constant because it is impossible to predict. However, the variability in the measurement of Hb level change was reduced by standardizing the timing of the postpartum blood test, performed at day 2, which reflects well the blood loss of the first 24 hours postpartum. In addition, the sensitivity analysis among women with prepartum Hb level measured within the two weeks before delivery provided similar results, which supports the validity of our results.

Defining UPPBL is a challenge. From previous studies that used peripartum change in Hb level or Ht value to define PPH, we decided on a ≥ 2g/dL peripartum change in Hb level to define UPPBL. These studies suggest that a ≥ 2 g/dL change in Hb level corresponds to ≥ 500 mL blood loss defining PPH [2,6,13,17,18,20] and that severe PPH (blood loss ≥ 1000 mL) may be defined by a ≥ 10% peripartum change in Ht value. We found different incidences of UPPBL depending on the definition, which probably corresponds to the degree of severity, although the risk factors for UPPBL according to these various definitions were comparable. The relevance of the 2-g/dL threshold for change in Hb level to define UPPBL is further supported by our finding of a median change in peripartum Hb level of 2.4 g/dL IQR [1.5–3.3,] with corresponding median blood loss of 600 mL IQR [500–800] at bag removal, for women with PPH.

Only two retrospective studies of UPPBL were found in literature [5,6], both are of small sample size and have methodological issues, but they report incidences similar to what we found with our main definition.

We were not able to directly evaluate medium- and long-term adverse consequences of UPPBL. Previous studies have shown adverse consequences of untreated postpartum anaemia, even if moderate (Hb ≤ 11.5 g/dL), on maternal and child health, as well as on mother–infant interactions up to 9 months after delivery [9,25]. In our study, more than half of the women with UPPBL (56.5% of UPPBL, 6.5% of deliveries) also had postpartum anaemia defined by Hb level ≤ 10 g/dL at day 2 postpartum. When we restricted the analysis to these women, the risk factors identified were comparable to those found with the main analysis.

The potential adverse consequences of UPPBL justify the attempt to characterize this entity as well as the circumstances and risk factors associated with it. Because all women were monitored with a collector bag in our study, our principal hypothesis explaining that some abnormal blood losses are undiagnosed is that some women have persistent bleeding, probably of a moderate and perhaps discontinuous flow, after the close monitoring period. Indeed, there are discrepancies between the classical definition of PPH [1,4], which refers to blood loss over the first 24 hours after delivery, and the clinical reality of the diagnosis established during the 2-hour close monitoring period in the delivery room, especially when the blood loss is measured in a collector bag that is usually removed at 15 to 30 min post-delivery. After the collector bag has been removed, surveillance may be less effective because the medical team has concluded that the blood loss is not excessive. This persistent bleeding could be caused by perineal laceration, uterine hypotonia or coagulopathy. This hypothesis is consistent both with the risk factors we identified and the fact that the collector bag was removed earlier in women with UPPBL, reflecting the birth attendant’s judgement of physiological bleeding. A blood suffusion of low abundance not requiring (in the view of the caregivers) any additional suture or further medical intervention is not uncommon after episiotomy and may explain UPPBL if persistent.

Our analysis also shows that the risk of UPPBL was increased twofold for women of Asian geographical origin, but their risk of PPH did not differ from that for women with other geographical backgrounds. An association between the Asian geographical origin and PPH was previously described in studies with PPH defined by biological criteria or based on International Classification of Diseases 9 codes [20,26,27]. We suspect that these types of definitions select a combination of PPH and UPPBL cases, which explains the association found. Indeed, other studies with PPH defined clinically did not report such an association [28]. The reason why women of Asian geographical origin may be more exposed to moderate but prolonged excessive postpartum bleeding needs further investigation.

Relying on the clinical diagnosis of postpartum anaemia symptoms is probably insufficient because those signs are often scarce or absent, except for severe cases. The biological diagnosis is also challenging given the current trend of early postpartum discharge, before day 2; moreover, a systematic blood count after delivery is not recommended by professional societies because some studies reported increased transfusion rate and low benefit/cost ratio in the short-term; however, the long-term cost/benefit balance of this systematic screening, considering the consequences for both the mother and the child, has not been evaluated [29,30]. Alternatively, postpartum screening could be targeted to women with risk factors for UPPBL. Considering the high frequency of UPPBL in our study and its potential adverse consequences, another option might be systematic iron supplementation in all women after delivery.

In our study, UPPBL appears to be frequent and have specific risk factors. A first implication of these findings for clinical practice is to emphasize the need for careful and prolonged surveillance of blood loss in the postpartum period, even after discharge from the labour ward and in particular with risk factors. Another implication is the need to define the optimal strategy to diagnose UPPBL. Further studies are needed to confirm our results and then evaluate the short- and long-term impacts of systematic or targeted screening for and treatment of postpartum anaemia.

## Supporting information

S1 Table Characteristics of women included and not included in the multivariate analysis. (DOCX)

S2 Table Multivariate analysis of association of women's characteristics with different definitions of UPPBL. (DOCX)

S3 Table Sensitivity analysis, multivariate analysis of risk factors for UPPBL and PPH among women who had a blood test within two weeks before delivery, (n = 1226). (DOCX)
