# Supplementary material for: Undiagnosed abnormal postpartum blood loss: Incidence and risk factors
Source: PLoS One. 2018 Jan 10;13(1):e0190845. doi: 10.1371/journal.pone.0190845 (PMC5761868; doi:10.1371/journal.pone.0190845)
Supplement: S1 Table — (DOCX) [file pone.0190845.s001.docx]

| Characteristics of women, labor and delivery | Population not included  n= 152 (4%) | | Analyzed population  n= 3765 (96%) | |
| --- | --- | --- | --- | --- |
| Asian geographic origin | 2 | (1.5) | 71 | (1.8) |
| Age (year) median, [IQR] | 31 | [28–35] | 30 | [26–34] |
| BMI^a^ (kg.m^-^²) median, [IQR] | 22.0 | [20.1–24.4] | 21.8 | [20.0–24.3] |
| Parity  Primipara  Multipara with no previous cesarean  Multipara with previous cesarean | 73  70  7 | (48.7)  (46.6)  (4.7) | 2110  1568  237 | (53.9)  (40.0)  (6.1) |
| History of clinical PPH | 2 | (1.5) | 79 | (2.0) |
| Preexisting chronic disease^b^ | 4 | (2.6) | 92 | (2.3) |
| Smoking before pregnancy | 28 | (21.2) | 1134 | (29.0) |
| Smoking during pregnancy | 15 | (13.9) | 635 | (16.2) |
| Polyhydramnios | 1 | (0.7) | 28 | (0.7) |
| Weight gain (kg)  median, [IQR] | 13 | [10–16] | 13 | [10–16] |
| Hypertensive disorder during pregnancy^c^ | 5 | (3.3) | 61 | (1.6) |
| Gestational age at delivery (weeks) [median, IQR] | 40 | [39–40] | 40 | [39–40] |
| Induction of labor | 34 | (22.4) | 750 | (19.1) |
| Total dose of oxytocin (mUI) median, [IQR] | 1347 | [488–3150] | 1075 | [450–2400] |
| Epidural anesthesia | 141 | (92.8) | 3799 | (97.0) |
| Hyperthermia during labor | 7 | (4.6) | 181 | (4.6) |
| Duration of labor (h) median, [IQR] | 3.2 | [2.1–4.8] | 3.5 | [2.3–5.0] |
| Duration of expulsive efforts (min) median, [IQR] | 15.5 | [8–25] | 13 | [7–23] |
| Mode of delivery  Spontaneous  Forceps  Vacuum  Spatula | 120  17  8  5 | (80.0)  (11.3)  (5.3)  (3.3) | 3195  265  276  179 | (81.6)  (6.8)  (7.1)  (4.6) |
| Type of perineal trauma  None  Episiotomy ± 1^st^ and 2^nd^ degree tear  1^st^ and 2^nd^ degree tear without episiotomy  3^rd^ and 4^th^ degree tear | 54  34  62  2 | (35.5)  (22.4)  (40.8)  (1.3) | 994  962  1757  52 | (26.4)  (46.7)  (25.6)  (1.4) |
| Retained placenta | 9 | (6.5) | 185 | (4.7) |
| Birth weight (g) median, [IQR] | 3430 | [3150–3750] | 3360 | [3090–3660] |
| Measured blood loss (ml) median, [IQR] | 100 | [60–300] | 100 | [60–300] |
| UPPBL^d^  PPH^e^ | 14  18 | (9.2)  (11.8) | 425  412 | (11.3)  (10.9) |

S1 Table. Characteristics of women included and not included in the multivariate analysis.

Data are no. (%) unless indicated.

IQR= interquartile range

*a: BMI: body mass index (weight (kg)/height² (m))*

*b:* *≥ 1 among high blood pressure, diabetes, and autoimmune disease*

*c:* *High blood pressure or preeclampsia during pregnancy*

*d: UPPBL: peripartum haemoglobin (Hb) level change of at least 2 g/dL without any clinical diagnosis of PPH.*

*e: PPH: blood loss ≥ 500 mL measured in the collector bag and/or any second-line treatment for PPH*
