# Supplementary material for: Undiagnosed abnormal postpartum blood loss: Incidence and risk factors
Source: PLoS One. 2018 Jan 10;13(1):e0190845. doi: 10.1371/journal.pone.0190845 (PMC5761868; doi:10.1371/journal.pone.0190845)
Supplement: S2 Table — (DOCX) [file pone.0190845.s002.docx]

| Characteristics of women, labour  and delivery | Definition 1^b^ /control  n=248 | p | Definition 2^c^ /control  n=614 | | p | Definition 3^d^  /control  n= 727 | | p |
| --- | --- | --- | --- | --- | --- | --- | --- | --- |
| Asian geographic origin | 1.7 (0.8-3.4) | 0.2 | 2.0 (1.1-3.7) | 0.02 | | 1.7 (1.0-3.1) | 0.06 | |
| Age (year) | 1.0 (1.0-1.0) | 0.4 | 1.0 (1.0-1.0) | 0.2 | | 1.0 (1.0-1.0) | 0.4 | |
| BMI*^e^* (/5 kg.m^-^²) | 1.0 (0.9-1.2) | 0.7 | 1.0 (0.8-1.1) | 0.7 | | 1.5 (1.3-1.6) | <0.001 | |
| Parity  Primipara  Multipara with no previous caesarean  Multipara with previous caesarean | 3.1 (1.9-4.9)  Ref.  3.1 (1.7-5.7) | <0.001 | 2.6 (1.9-3.4)  Ref.  2.5 (1.6-3.8) | <0.001 | | 2.1 (1.6-2.8)  Ref.  2.3 (1.6-3.4) | <0.001 | |
| History of clinical PPH | 0.9 (0.2-3.7) | 0.8 | 1.0 (0.4-2.4) | 0.9 | | 1.4 (0.7-2.9) | 0.7 | |
| Smoking during pregnancy | 0.6 (0.4-1.0) | 0.05 | 0.8 (0.6-1.1) | 0.2 | | 1.0 (0.8-1.3) | 0.8 | |
| Hypertensive disorder during^f^ pregnancy | 0.8 (0.3-2.6) | 0.7 | 0.7 (0.3-1.6) | 0.3 | | 0.9 (0.4-1.8) | 0.7 | |
| Gestational age at delivery (WG) | 1.1 (1.0-1.3) | 0.1 | 1.0 (0.9-1.1) | 0.3 | | 1.1 (1.0-1.2) | 0.1 | |
| Induction of labour | 1.0 (0.7-1.4) | 0.9 | 1.2 (0.9-1.5) | 0.3 | | 1.1 (0.8-1.4) | 0.5 | |
| Duration of labour (/2h) | 1.1 (1.0-1.2) | 0.03 | 1.1 (1.1-1.2) | <0.001 | | 1.1 (1.0-1.2) | 0.002 | |
| Duration of expulsive efforts (/10 min) | 0.9 (0.8-1.1) | 0.1 | 1.0 (0.9-1.1) | 0.6 | | 1.0 (0.9-1.1) | 0.7 | |
| Total dose of oxytocin (mUI)  0  0-460  461-1650  >1650 | Ref.  1.3 (0.8-2.1)  1.3 (0.8-2.0)  1.7 (1.1-2.6) | 0.07 | Ref.  1.3 (1.0-1.8)  1.3 (1.0-1.8)  1.4 (1.0-1.9) | 0.1 | | Ref.  1.3 (1.0-1.8)  1.4 (1.0-1.8)  1.4 (1.0-1.9) | 0.07 | |
| Hyperthermia during labour | 1.2 (0.7-1.9) | 0.6 | 1.1 (0.7-1.6) | 0.6 | | 1.0 (0.7-1.5) | 0.9 | |
| Mode of delivery  Spontaneous  Forceps  Vacuum  Spatula | Ref.  2.3 (1.4-3.7)  1.3 (0.7-2.3)  2.1 (1.3-3.4) | <0.001 | Ref.  2.8 (1.9-4.1)  1.3 (0.8-2.0)  2.2 (1.4-3.2) | <0.001 | | Ref.  2.6 (1.8-3.9)  1.3 (0.9-1.9)  2.3 (1.6-3.5) | <0.001 | |
| Type of perineal trauma  None  Episiotomy ± 1^st^ and 2^nd^ degree tear  1^st^ and 2^nd^ degree tear without episiotomy  3^rd^ and 4^th^ degree tear | Ref.  2.8 (1.8-4.4)  1.0 (0.6-1.6)  3.2 (1.2-8.9) | <0.001 | Ref.  3.0(2.2-4.1)  1.1 (0.8-1.4)  2.1 (0.9-4.8) | <0.001 | | Ref.  3.2 (2.4-4.3)  1.3 (1.0-1.6)  2.2 (1.0-4.8) | <0.001 | |
| Retained placenta | 2.5 (1.4-4.4) | 0.002 | 2.2 (1.4-3.4) | <0.001 | | 1.9 (1.2-2.9) | 0.003 | |
| Birth weight (/500g) | 1.0 (0.9-1.3) | 0.6 | 1.0 (0.9-1.2) | 0.5 | | 1.1 (1.0-1.2) | 0.1 | |

S2 Table. Multivariate analysis^a^ of association of women's characteristics with different definitions of UPPBL

Data are adjusted odds ratios (95% confidence interval)

***a: Multivariate model including all the variables listed in the table and the maternity unit***

*b: Definition 1: Peripartum Hb level change ≥2 g/dL and Hb level ≤10g/dL*

*c: Definition 2: Peripartum change in haematocrit value ≥ 5%*

*d: Definition 3: Blood loss ≥ 500 mL calculated by (peripartum Ht change) x total blood volume (TBV))/0.35, where TBV (mL) = weight (in kg) x 65 x 1.4*

*e: BMI: body mass index (weight (kg)/height² (m))*

*f: High blood pressure or preeclampsia during pregnancy*
