# Supplementary material for: Undiagnosed abnormal postpartum blood loss: Incidence and risk factors
Source: PLoS One. 2018 Jan 10;13(1):e0190845. doi: 10.1371/journal.pone.0190845 (PMC5761868; doi:10.1371/journal.pone.0190845)
Supplement: S3 Table — (DOCX) [file pone.0190845.s003.docx]

S3 Table. Sensitivity analysis, multivariate analysis^a^ of risk factors for UPPBL and PPH among women who had a blood test within two weeks before delivery, (n=1226)

| Characteristics of women, labour and delivery | UPPBL  /control  aOR^a^ (_95%_CI) | p | PPH  /control  aOR^a^ (_95%_CI) | p |
| --- | --- | --- | --- | --- |
| Asian geographic origin | 2.5 (1.1-5.6) | **0.03** | 1.3 (0.4-4.0) | 0.6 |
| Age (year) | 1.0 (1.0-1.0) | 0.7 | 1.0 (1.0-1.0) | 0.6 |
| BMI^b^ (/5 kg.m^-^²) | 0.8 (0.7-1.0) | 0.1 | 0.9 (0.7-1.1) | 0.3 |
| Parity  Primipara  Multipara with no previous caesarean  Multipara with previous caesarean | 2.7 (1.5-4.7)  Ref.  4.5 (2.3-8.9) | **<0.001** | 1.3 (0.7-2.3)  Ref.  1.7 (0.7-3.7) | 0.4 |
| History of clinical PPH | 1.0 (0.3-4.1) | 1.0 | 5.1 (1.8-14.7) | **0.002** |
| Smoking during pregnancy | 0.6 (0.4-1.1) | 0.1 | 0.8 (0.4-1.4) | 0.4 |
| Hypertensive disorder during pregnancy^c^ | 0.7 (0.2-2.2) | 0.5 | 2.5 (0.9-6.8) | 0.2 |
| Gestational age at delivery (weeks’ gestation) | 1.0 (0.8-2.0) | 0.2 | 1.3 (1.1-1.6) | **0.002** |
| Induction of labour | 1.3 (0.8-2.0) | 0.2 | 1.4 (0.8-2.2) | 0.2 |
| Duration of labour (/2 hr) | 1.1 (0.9-1.2) | 0.2 | 1.0 (0.9-1.1) | 0.6 |
| Duration of expulsive efforts (/10 min) | 0.9 (0.7-1.0) | 0.1 | 1.1 (0.9-1.3) | 0.4 |
| Total dose of oxytocin (mUI) during labour  0  0-460  461-1650  >1650 | Ref.  1.2 (0.7-2.2)  1.3 (0.7-2.1)  1.3 (0.7-2.3) | 0.8 | Ref.  0.9 (0.4-1.7)  0.7 (0.3-1.3)  1.5 (0.8-2.9) | **0.03** |
| Hyperthermia during labour | 1.8 (0.8-2.1) | 0.2 | 0.7 (0.3-1.6) | 0.4 |
| Mode of delivery  Spontaneous  Forceps  Vacuum  Spatula | Ref.  2.6 (1.3-5.0)  1.1 (0.5-2.4)  2.5 (1.4-4.3) | **<0.001** | Ref.  4.3 (2.1-8.6)  1.9 (1.0-3.7)  4.4 (2.2-8.5) | **<0.001** |
| Type of perineal trauma  None  Episiotomy ± 1^st^- and 2^nd^-degree tear  1^st^- and 2^nd^-degree tear without episiotomy  3^rd^- and 4^th^-degree tear | Ref.  3.8 (2.0-7.0)  1.1 (0.6-2.0)  5.3 (1.3-22.3) | **<0.001** | Ref.  5.2 (2.3-11.6)  2.9 (1.3-6.2)  8.3 (1.7-39.9) | **<0.001** |
| Retained placenta | 4.0 (1.9-8.5) | **<0.001** | 9.6 (4.7-19.5) | **<0.001** |
| Birth weight (/500 g) | 1.0 (0.8-1.3) | 0.7 | 1.0 (0.7-1.3) | 0.9 |

*a: Multivariate model including all the variables listed in the table and the maternity unit*

*b: BMI: body mass index (weight (kg)/height² (m))*

*c:* *Gestational hypertension or preeclampsia during pregnancy*
